# Clinical effects of durability of immunosuppression in virologically suppressed ART-initiating persons with HIV in Latin America. A retrospective cohort study

**DOI:** 10.1016/j.lana.2021.100175

**Published:** 2022-01-13

**Authors:** Yanink Caro-Vega, Peter F. Rebeiro, Bryan E. Shepherd, Pablo F. Belaunzarán-Zamudio, Brenda Crabtree-Ramirez, Carina Cesar, Paula Mendes Luz, Claudia P. Cortes, Denis Padget, Eduardo Gotuzzo, Catherine C. McGowan, Juan G. Sierra-Madero

**Affiliations:** a Instituto Nacional de Ciencias Médicas y Nutrición Salvador Zubirán (INCMNSZ), Mexico City, Mexico; b Vanderbilt University School of Medicine, Nashville, TN, USA; c Fundación Huésped, Buenos Aires, Argentina; d Instituto Nacional de Infectología Evandro Chagas, Río de Janeiro, Brasil; e Fundación Arriarán y Universidad de Chile, Santiago, Chile; f Instituto Hondureño de Seguridad Social, Tegucigalpa, Honduras; g Universidad Peruana Cayetano Heredia, Lima, Perú

**Keywords:** HIV, Sustained Virologic Response, Latin America, cumulative low CD4 counts, AIDS defining and non-AIDS defining events

## Abstract

**Background:**

Clinical outcomes are rarely studied in virologically suppressed people living with HIV (PWH) and incomplete CD4 recovery. To explore whether time living with severe immunosuppression predict clinical outcomes better than baseline or time updated CD4, we estimated the association between cumulative percentage of time with CD4 <200 cells/µL during viral suppression (VS) (%t_CD4<200_), and mortality and comorbidities during 2000-2019.

**Methods:**

In a retrospective cohort analysis, we followed PWH initiating ART in Latin America from first VS (HIV-RNA<200 copies/µL) to death, virological failure or loss to follow-up. We fit Cox models to estimate risk of death and/or AIDS-defining and serious non-AIDS-defining events (ADE and SNADE -cancer, cardiovascular, liver, and renal diseases) by %t_CD4<200_ (continuous variable). We predicted survival probabilities for each event and calculated risks of hypothetical cases of different %t_CD4<200_.

**Findings:**

In 8,369 patients with 34·9 months of follow-up (median, IQR: 16·7, 69·1), 4,274 (51%) started ART with CD4<200 cells/µL. Median %t_CD4<200_ was 0% (IQR: 0, 15%). We identified 195 (2·3%) deaths and 584 (7·2%) patients with ADE/SNADE. For an increased %t_CD4<200_ of 15% (e.g., 15% vs. 0%), the adjusted relative hazard (aHR) of death was 1·27 (95% confidence interval [CI]: 1·19 – 1·35), of ADE/SNADE was 1·13 (95%CI: 1·09 – 1·17), of SNADE was 0·96 (95%CI: 0·89 – 1·02) and of death/ADE/SNADE was 1·11 (95%CI: 1·07 – 1·14). Estimates were similar after adjusting for time updated CD4 count.

**Interpretation:**

In virologically suppressed PWH, increased time living with severe immunosuppression had an increased risk of death and ADE/SNADE in this Latin American cohort, independently of time updated CD4 count.

**Funding:**

This work was supported by the NIH-funded Caribbean, Central and South America network for HIV epidemiology (CCASAnet, U01AI069923), a member cohort of the International Epidemiologic Databases to Evaluate AIDS (leDEA). This award is funded by the following institutes: Eunice Kennedy Shriver National Institute Of Child Health & Human Development (NICHD), National Cancer Institute (NCI), National Institute Of Allergy And Infectious Diseases (NIAID), National Institute Of Mental Health (NIMH), the National Heart, Lung, and Blood Institute (NHLBI), the National Institute on Alcohol Abuse and Alcoholism (NIAAA), the National Institute of Diabetes and Digestive and Kidney Diseases (NIDDK), and the Fogarty International Center (FIC). Specific funding was provided from the Fogarty International Center (FIC) for lead author, Yanink Caro-Vega, for the Fogarty-IeDEA Mentorship Program (FIMP).

**Research in context**

**Evidence before this study**

People living with HIV (PWH) and incomplete immune recovery after initiating antiretroviral therapy (ART) have higher risk of comorbidities and death, despite successful virologic suppression. Their risk of mortality has been reported from one to four times higher, and from 0·96 to 2·70 for developing AIDS, when compared with PWH with non-discordant immune responses. There is no information about the impact of incomplete immune recovery on risk for non-AIDS-defining events. In Latin America we have reported that 50% of patients initiate ART with CD4 counts <200 cells/µL, and 25% of them do not reach levels above 200 cells/µL five years after treatment initiation. Some studies have shown association between lower cumulative CD4 counts and death and comorbidities, after adjusting for viral load. We were interested in these associations only under periods of virologic suppression to avoid the potential influence of poor adherence or ART interruptions.

**Added value of this study**

In this large cohort of PWH receiving care in Latin America who were virally suppressed on ART, we found that the percentage of time they lived with CD4<200 cells/µL was strongly associated with mortality and clinical events, even after controlling for updated CD4 cell counts. Patients starting ART with CD4<200 cells/µL had a median of 38% (IQR: 0 – 100%) of the time with CD4 counts lower than 200 cells/µL at 12 months after first VS, 16% (IQR: 0 – 57%) at 24 months, and 10% (IQR: 0 – 28%) at 48 months. We identify that PWH virally suppressed on ARTs that live severely immunosuppressed with greater percentage of time have a significantly increased risk of mortality (27%, 95%CI 19%-35%) and of serious non-AIDS-defining events (11%, 95%CI 7%-14%) even after adjusting for updated CD4 counts.

**Implications of all the available evidence**

Despite current recommendations to perform less CD4 monitoring in virally suppressed patients, our study supports the importance of continued CD4 count monitoring to identify patients at higher risk of developing AIDS-defining events or serious non-AIDS-defining events, in particular in those patients with CD4 counts lower than 200 cells/µL.

Alt-text: Unlabelled box

## Introduction

People living with HIV (PWH) receiving virally suppressive combination antiretroviral therapy (ART) with incomplete immunologic recovery are at higher risk of AIDS-defining events (ADE) and death^1^ in comparison to people with complete immunologic recovery.^2^^,^^3^ The observed risk of mortality among PWH and discordant immune responses, defined as “a failure to increase CD4 count on ART despite successful virological control”,^1^ has been shown to be one to four times higher than that of their counterparts with adequate immune recovery^1^ and nearly two times higher in a limited resource setting.^4^ The relative risk of ADE for people with immune discordant response may range from 0·96 to 2·70, but has been less frequently studied.^1^ Only one study explored the association with serious non-AIDS-related events, and observed no significant differences between participants with discordant (6%) and non-discordant (5%) responses.^1^

Low CD4 count and CD4/CD8 ratio^5^ in virally suppressed patients have been identified as markers of general persistent inflammation which might be related to the increased risk of death^6^ and comorbidities.^1^^,^^7^ Males, people initiating ART late, and older PWH are at increased risk for incomplete immune response, according to results from a single HIV cohort.^2^ We have observed in previous studies in the Central and South America network for HIV epidemiology (CCASAnet) cohort in Latin America that almost 50% of patients initiate ART with CD4 counts <200 cells/ µL,^8^ nearly 25% of them do not reach levels above 200 cells/µL five years after treatment initiation, and a high proportion of deaths can be attributed to initiating ART with CD4 <200 cells/ µL.^9^ When examining factors associated with mortality, our group has usually considered CD4 at ART initiation or time-updated CD4 measurements in these analyses. In this study we tested the hypothesis that time living with severe immunosuppression (CD4<200 cells/ µL) may be a better predictor of risk for clinical outcomes than baseline CD4 or time-updated CD4 measurements.

Few studies have analyzed the potential impact of time exposed to low CD4 counts in virally suppressed patients receiving ART on the incidence of comorbidities. Cumulative CD4 counts were used as a predictor of acute myocardial infarction and mortality compared to VACS index, viremia-years, and time-updated single measurements of viral load and CD4, and was found associated with acute myocardial infarction to the lowest CD4 count level.^10^ In another study, a higher CD4 count-year, a time-varying measure of immunologic response, was found associated with a lower risk of cancer incidence.^11^ None of these studies was restricted to patients with viral suppression (VS), although the viral load covariate was used in the fitted models. While there are no effective interventions recommended to increase CD4 counts in PWH receiving ART, information from cohort studies may contribute to design better monitoring practices for early diagnosis and timely treatment of non-AIDS related events. Thus, our aim was to estimate the association of cumulative percentage of time with CD4 <200 cells/µL on mortality and AIDS- and non-AIDS-related comorbidities among ART-initiators with VS in the CCASAnet cohort during 2000-2019.

## Methods

### Study Population

The CCASAnet^#^ cohort was established to analyze the characteristics of the HIV epidemic and clinical care in Latin America and the Caribbean.^12^ We used cohort data updated in April 2020, including information from sites in Argentina, Brazil, Chile, Honduras, Mexico and Peru where viral load and CD4 measurements are systematically collected for routine patient care. We selected PWH (≥18 years) initiating ART between 2000-2017, with documented VS during the first 12 months of ART initiation and followed-up until 2019. Viral suppression was defined as maintaining repeated viral load measurements lower than 200 copies/mL, without any occurrence of virological failure. Patients were followed in the study since the first date of VS until documented virological failure (one viral load measurement >1000 copies/mL or two consecutive viral load measurements >200 copies/mL), death, or lost to follow-up. A patient was considered lost to follow-up if their last visit date was more than one year before the closure date of the cohort. If an individual's last visit or death occurred less than one year after the last viral load available, and that viral load was <200 copies/mL, follow-up ended at the time of last visit or death. If their final viral load available was ≥200 copies/mL, follow-up ended at their last viral load measurement <200 copies/mL (Supplementary Figure 1). Patients with no available follow-up based on lack of CD4 counts for more than one year after first VS were excluded from the study. If there was a period of ≥1 year with no viral load or CD4 measurement, the patient was censored at their last measurement. We classified patients in two groups according to their CD4 count at ART initiation as patients initiating with a CD4 <200 cells/µL (CD4<200) and patients initiating with a CD4 ≥200 cells/µL (CD4≥200). CD4 count at ART initiation was chosen as the closest measure in a window of -90 and +30 days around ART initiation date.

### Outcomes and variables of interest

We organized a dataset with the complete CD4 count history of patients selected while viral suppression was documented. We estimated the cumulative percentage of time with CD4<200 (%t_CD4<200_) at each date. We assumed that a CD4 count was the same until the next available measurement.

We were interested in the following outcomes: 1) death; 2) ADE or SNADE; 3) any event (death, ADE or SNADE); 4) SNADE. Deaths reported were those occurring a maximum of 365 days after the last viral load <200 copies/mL available. For SNADE, we included non-AIDS-defining cancers, cardiovascular, liver and renal disease diagnoses reported during follow-up. Events were diagnosed during routine care and registered prospectively in the medical record and case report forms designed for the CCASAnet cohort. Definitions for these events are detailed in a previous publication from our group.^13^ AIDS related events were reported according to accepted definitions.^14^

### Statistical analyses

We described the time elapsed under viral suppression, percentage of time with CD4 below 200 (%t_CD4<200_), sex, age at ART initiation, level of education, HIV acquisition risk, time updated CD4 count, calendar year of ART initiation, and site; based on CD4 count at ART initiation (<200 or ≥200). The number of events (deaths, ADE and SNADE) was established in each group and crude rates were estimated. We used a logistic regression model to identify characteristics of patients associated with the proportion of the patient's time that CD4 count was below 200 cells/ µL. The outcome in this model was the proportion of time at the end of follow-up that the patient had CD4 <200; logistic regression (which is usually used for binary data) can be fit to and has been recommended by others for the analysis of proportions.^15^^,^^16^ We used Cox models to estimate the risk of event using three alternative time-varying predictors: %t_CD4<200_, the time-updated CD4, and both predictors.^17^ We adjusted by sex [assigned at birth as male or female; we did not have data on transgender individuals], age [continuous], probable HIV acquisition risk [men who have sex with men, heterosexual, other: injecting drug users, perinatal, transfusion, non-hemophilia related; and unknown], education level [primary, secondary, post-secondary, unknown], site, and calendar-year of ART initiation [continuous] as covariates. For estimation, we built a long format data set with start and end dates by record according to the CD4 count dates available for each patient; each person-period began with a CD4 measurement date. At each person-period, the cumulative percentage of time with CD4 count under 200 cells/µL up until that date was estimated, and the status of death or AIDS-defining events and serious non-AIDS defining events during the period was evaluated. We tested the proportional hazard assumption of the models using the *cox.zph* test in R, which is based on Schoenfeld residuals. To compare the fit of the models we used their Akaike Information Criterion (AIC).^18^ From the fitted models, we predicted survival probabilities for each event for the hypothetical cases of patients spending 0%, 50%, and 90% of their time with CD4 counts below 200 cells/ µL, and the updated CD4 counts of 200, 350 and 500. We fit similar Cox models to estimate the risk of each of our outcomes: death; ADE/SNADE; death/ADE/SNADE; and SNADE. For the ADE/SNADE or SNADE, death is a competing risk; people who died were censored at the time of death, and these models are therefore estimating cause-specific hazards.^19^ serious non-AIDS defining events using %t_CD4<200_ as a predictor. In all models that included SNADEs as the event or part of the composite event, we excluded Honduras data because serious non-AIDS events were not reported at that site. Analyses were performed using R Version 3.6.0

### Role of the funding source

The funders had no role in the design of the study; in the collection, analyses, or interpretation of data; or in the writing of the report.

## Results

**Figure 1:**
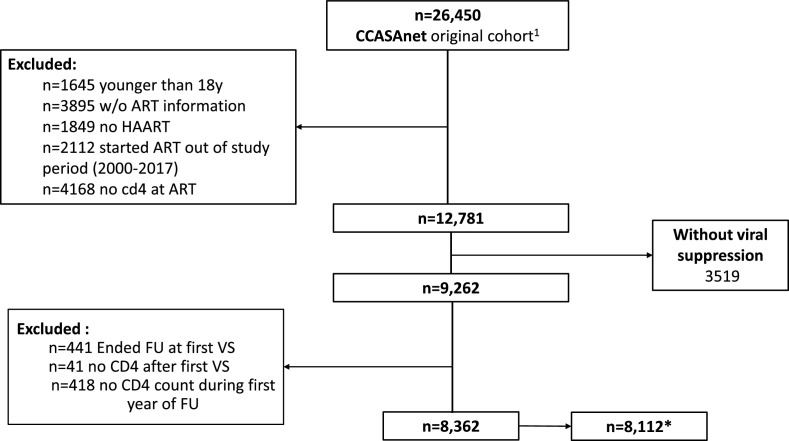
Flow chart of patients included in the study. Note: ^1^ Patients from Haiti (n=20,858) were excluded because VL was not systematically collected over most of the study period. CD4 count at ART initiation was defined as closest value to ART initiation within 90 days before and 30 days after. VS: Viral suppression, patients reaching viral loads under 200copies/mL any time within the first 12 months of ART initiation. *For analysis with AIDS and serious non-AIDS defining events in the outcome, we excluded Honduras.

We included 8,362 patients that achieved VS (Figure 1) at a median of 3·43 months (IQR: 2·6 – 5·4) after ART initiation. These patients were then followed while virally suppressed for a median of 34·9 months (IQR: 16·7 – 69·1). Patient characteristics according to baseline CD4 count and overall are shown in Table 1. In all, 78% of patients included were male, with a median age at ART initiation of 33·9 years (IQR: 27·6 – 42·3), 90% with secondary level of education or higher, and mostly with men who have sex with men HIV acquisition risk (50%). A total of 4,274 (51%) of patients had CD4 count at ART initiation lower than 200 cells/µL.

**Table 1:** Characteristics of the study population with viral suppression documented after ART initiation by group of CD4 count.

Characteristic	CD4 at ART <200 N=4,274 (51%)	CD4 at ART ≥200 N=4,088 (49%)	Complete population N=8,362	p-value
Male, n(%)	3,368 (79%)	3,158 (77%)	6,526 (78%)	0.09
Age at ART initiation in years, median (IQR)	35.6 (29.1 – 43.7)	32.1 (26.1 – 40.7)	33.9 (27.6 – 42.3)	<0.001
Education level, n(%)				<0.001
Less than Primary	485 (11%)	426 (10%)	911 (10%)	
Secondary	2120 (49%)	1879 (46%)	3999 (48%)	
University	1260 (29%)	1433 (35%)	2693 (32%)	
Unknown	409 (9%)	350 (8%)	759 (9%)	
Probable HIV acquisition risk, n(%)				<0.001
Men who have Sex with Men	1931 (45%)	2257 (55%)	4188 (50%)	
Heterosexual	2020 (47%)	1528 (37%)	3548 (42%)	
Other	37 (<1%)	49 (1%)	80 (1.0%)	
Unknown	286 (6.6%)	254 (6%)	540 (6.4%)	
CD4 at ART initiation, cells/mL	74 (31 – 131)	328 (261 – 445)	193 (71 – 324)	<0.001
CD4 at Follow-up initiation, cells/mL	157 (84 – 242)	432 (325 – 588)	285 (152 – 447)	<0.001
ADE at ART initiation	2211 (60%)	593 (18%)	2804 (41%)	<0.001
Site				<0.001
Argentina	201 (4.7%)	283 (7%)	484 (6%)	
Brazil	988 (23%)	1487 (36%)	2475 (30%)	
Chile	585 (14%)	602 (15%)	1187 (14%)	
Honduras	180 (4.2%)	66 (1.6%)	246 (3%)	
Mexico	730 (17%)	511 (12%)	1241 (15%)	
Peru	1590 (37%)	1139 (28%)	2985 (33%)	
Calendar year of Start of follow-up	2011 (2007 – 2014)	2013 (2009 – 2015)	2012 (2008 – 2014)	<0.001
Time since ART initiation to start of follow-up (first VS), months	3.5 (2.8 – 5.6)	3.3 (2.4 – 5.1)	3.4 (2.6 – 5.4)	<0.001
Total time of follow-up, months (under viral suppression)	38.8 (17.9 – 77.6)	31.2 (15.5 – 61.1)	34.9 (16.7 – 69.1)	<0.001

A total of 195 (2·3%) deaths were reported, occurring in a median of 25·2 months (IQR: 11 – 50) after start of follow-up (first VS), with a crude incidence of 5·84 deaths per 1000-person years; 135 (69% of deaths) occurred among patients starting ART with CD4 <200 cells/µL. Among 8,116 patients, excluding Honduras, we identified 584 patients with ADE or SNADE, of whom 209 were SNADE (36%). Distribution of specific comorbidities included in ADE and SNADE group is shown in Supplementary Table 1. The median time from first VS to event among those who experienced an ADE or a SNADE event was 10·5 months (IQR: 3·1 – 29·4) and the crude incidence for this outcome was 19·1 events per 1000-person years. A total of 704 (8·7%) patients had any event (death, ADE or SNADE): 71 had ADE or SNADE and death, 513 only ADE or SNADE and 120 had death alone; 466 (66%) of patients with any event, were patients initiating ART with CD4 <200 cells/µL.

The median %t_CD4<200_ under suppression after first VS was 0% (IQR:0 – 15%). At the end of the study period, 69% of patients spent no time after VS with CD4 <200 cells/µL; 21% of patients spent >15% of their time after VS with CD4 <200 cells/µL. Percentages differed according to CD4 count at ART initiation (< vs. ≥200 cells/µL) and the time in follow-up (Supplementary Figure 2). Patients starting ART with CD4 <200 cells/µL had a median %t_CD4<200_ of 38% (IQR: 0 – 100%) 12 months after first VS, 16% (IQR: 0 – 57%) at 24 months, and 10% (IQR:0 – 28%) at 48 months. In contrast, patients starting ART with CD4 ≥200 had a median %t_CD4<200_ of 0% at all times.

Being male, older, initiating ART in an earlier calendar year, being from Honduras, having a CD4 <200 cells/µL at ART initiation, and recent calendar year of follow-up were associated with more percentage time spent with CD4 <200 cells/µL during VS (Supplementary Table 2). Specifically, we found that the odds of having CD4 <200 over follow-up were 57% higher for males than females, 18% higher for every ten-year increase in age, and 162% higher for those starting ART with a CD4 <200 cells/ µL compared to those starting ART with a CD4 ≥200 cells/ µL. The odds of having CD4 <200 cells/µL decreased by 21% among men who have sex with men compared to those with heterosexual HIV acquisition risk, and decreased 18% among those with university education compared to primary level.

**Figure 2:**
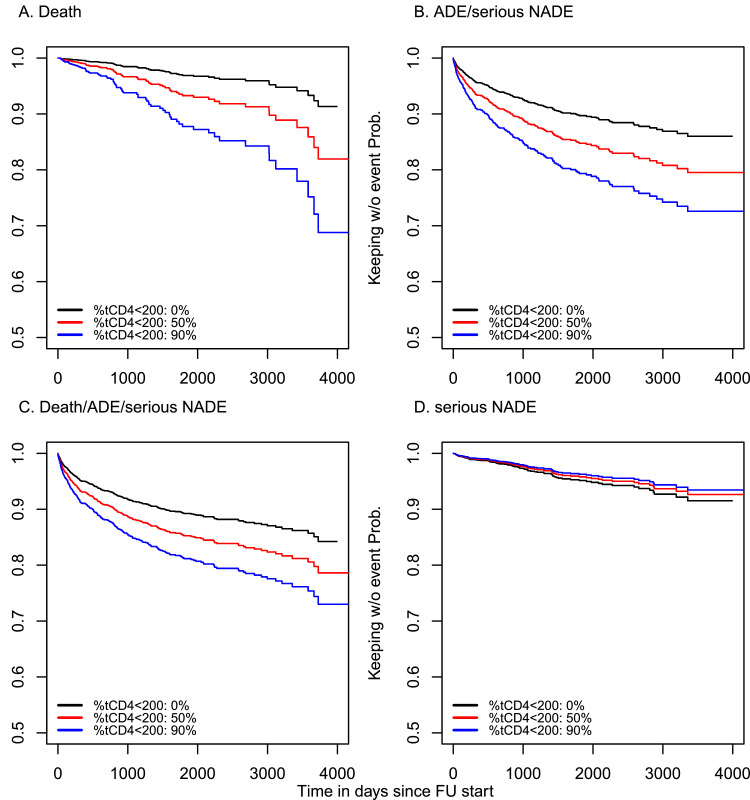
Survival probability for death, AIDS-defining and/or serious non-AIDS defining events by percentage of time with CD4 count lower than 200. Note: Predicted survival from Cox model for death, clinical event and any event. Plots were predictions for males, MSM as probable HIV acquisition risk, age 40oy, less than 200 at ART initiation, scholar level Secondary, year of follow-up start 2012 and brazil. Panels B, C and D exclude Honduras.

Percentage of time spent with CD4 <200 in patients (%t_CD4<200_) with virologic suppression was a strong predictor of outcomes in this population. For an increase in %t_CD4<200_ of 15% (e.g., 15% vs. 0%), the adjusted relative hazard (aHR) of death was 1·27 (95% confidence interval [CI]: 1·19 – 1·35), of ADE or SNADE was 1·13 (95% CI: 1·09 – 1·17), and of the composite outcome of death, ADE, or SNADE was 1·11 (95% CI: 1·07 – 1·14) (Table 2). Estimates for 15% increases in %t_CD4<200_ were similar when time-updated CD4 count was included in the model: aHR=1·09, (95%CI: 1·05 – 1·14) for ADEs or SNADEs; and aHR=1·08, (95%CI: 1·04 – 1·12) for the composite outcome; but different for death alone: aHR=1·05, (95%CI: 0·97 – 1·14). Adjusted hazard ratios for the models that only included time updated CD4 count are also shown in Table 2. Based on the Cox model using only %t_CD4<200_, Figure 2 shows the predicted survival probabilities curves for death, ADE and SNADE, any event, and only SNADE, for hypothetical cases of 0%, 50%, and 90% of time of follow-up under viral suppression spent with CD4 counts lower than 200 cells/ µL.

**Table 2:** Hazard ratio for death, AIDS-defining and serious non-AIDS-defining events using the models including percentage of time with CD4<200, updated CD4 count and both predictors.

		Death		ADEs or SNADE		Any event (Death, ADE, or SNADE)		SNADE only	
		HR	95%CI	HR	95%CI	HR	95%CI	HR	95% CI
**Percentage of time as predictor of interest**	Percentage of time with CD4<200 1								
	15% vs 0%	1.27	1.19 – 1.35	1.13	1.09 – 1.17	1.11	1.07 – 1.14	0.96	0.89 - 1.02
	50% vs 0%	2.20	1.78 – 2.71	1.52	1.35 – 1.71	1.40	1.26 – 1.55	0.86	0.68 - 1.09
	90% vs 0%	4.13	2.82 – 6.04	2.12	1.72 – 2.63	1.83	1.52 – 2.21	0.77	0.50 - 1.16
**Updated CD4 count as predictor of interest**	Updated CD4 count^2^								
	350 vs 200	0.47	0.41 – 0.55	0.77	0.72 – 0.84	0.85	0.79 – 0.90	1.00	0.91 – 1.11
	500 vs 200	0.22	0.17 – 0.30	0.60	0.52 – 0.70	0.72	0.63 – 0.81	1.00	0.82 – 1.23
**Percentage of time and updated CD4 count as predictors of interest**	Percentage of time with CD4<200								
	15% vs 0%	1.05	0.97 – 1.14	1.09	1.05 – 1.14	1.08	1.04 – 1.12	0.95	0.88 – 1.02
	50% vs 0%	1.19	0.92 – 1.53	1.34	1.17 – 1.53	1.30	1.15 – 1.46	0.84	0.65 – 1.08
	90% vs 0%	1.36	0.86 – 2.16	1.70	1.33 – 2.16	1.60	1.29 – 1.99	0.73	0.46 – 1.14
	Updated CD4 count								
	350 vs 200	0.51	0.42 – 0.61	0.85	0.79 – 0.93	0.92	0.85 – 0.98	0.97	0.86 – 1.09
	500 vs 200	0.26	0.18 – 0.37	0.73	0.61 – 0.86	0.84	0.73 – 0.97	0.94	0.75 – 1.18

All the models include Probable HIV acquisition risk, education level, CD4 at ART initiation group, calendar year of follow-up start and stratified by site. Models with ADE and SNADE in the outcome excluded Honduras site. Aikaike´s criterion for death in the three models: 2476, 2410, 2410. AIC for ADE and SNADE: 8158, 8161 and 8144. AIC for any event (death or clinical event) 10007, 10019 and 10003. AIC for SNADE was 2823, 2824, and 2824.

1 Percentage of time with CD4<200 was included in models as a continuous variable; the values 15%, 50% and 90% were compared to 0% to improve interpretation of the associations. These can be thought of as the adjusted relative hazards for 15%, 50%, and 90% increases in the percentage of time with CD4<200.

2 Similarly, updated CD4 count was also included in models as a continuous variable; the values 350 and 500 were compared to 200 for interpretation. These can be thought of as the adjusted relative hazards for a 150 (350 vs. 200) and 300 (500 vs. 200) cell/mm^3^ increase in CD4.

From the Cox model for SNADE as the only outcome, we did not find a statistical association with the %t_CD4<200_; estimates for %t_CD4<200_ of 15% vs. 0% was aHR: 0·96 (95%CI: 0·89 – 1·02) and for %t_CD4<200_ of 90% vs. 0% was aHR: 0·77 (95%CI: 0·50 – 1·16) (Table 2).

## Discussion

In this large cohort study of PWH in Latin America who were virally suppressed after ART initiation, we found that the percentage of time living with CD4 <200 cells/µL was strongly associated with mortality and clinical events, even after controlling for time-updated CD4 cell count. Male sex, older age, and initiating ART with a CD4 <200 cells/µL were associated with a higher percentage time with CD4<200 cells/µL during viral suppression. Our results are very relevant for our region, considering the persistent high frequency of late HIV diagnosis and ART initiation in our population,^19^, ^20^, ^21^ as these patients are at a higher risk of spending longer time with low CD4 counts. Moreover, this group is at a higher risk of death and developing ADE or SNADE, even when eventually achieving adequate immune responses.

Previous studies have shown an association between lower cumulative CD4 counts and death^10^ and cardiovascular events and cancers^10^^,^^11^ after adjusting for viral load. Other studies have shown similar results for specific comorbidities, most of them focused on AIDS-defining events. In our study, we included only periods of virologic suppression, which allowed us to focus on estimating the association of time with low CD4 counts with relevant outcomes, without the influence of fluctuations in viral load associated with poor adherence, or ART interruptions.^11^ Furthermore, in our study, the percent-time with CD4 <200 still remained predictive of ADE or SNADE after adjusting for updated CD4 counts, suggesting that time living with low CD4 counts, even months or years earlier, increases the risk of comorbidities regardless of current immune status.

As expected, we found that a low CD4 count at ART initiation was associated with longer periods of time with CD4 <200, and with higher risk of comorbidities. In this study, 51% of people started ART with CD4 count lower than 200 cells/ µL, spending a median of 36% of time during the first year after VS with CD4 <200 cells/µL. Previous reports indicated a high proportion of late presentation and late ART initiation in Latin America^20^, ^21^, ^22^ and their association with an increased prevalence of non-communicable diseases.^22^ People initiating ART late can achieve similar proportions of VS as people initiating earlier, but even after many years of ART, they generally have lower CD4 counts and less normalization of CD4/CD8 ratios, clinical markers that have been related to higher risks of death and comorbidities.^23^ Few studies in Latin America have been conducted to describe the prevalence of comorbidities among PWH, but an increasing trend of multimorbidity along time has been documented, especially among people older than 50 years of age,^22^^,^^24^ women, and people with low CD4 count nadir.^22^

Although we were not able to find a significant association between serious non-AIDS defining events alone and the percentage time with CD4 count lower than 200, it is important to highlight that the number of serious non-AIDS defining events was relatively small compared to the total number of events. Few studies have looked for an association between immune response and non-AIDS defining events,^1^ reporting a slightly higher proportion of non-AIDS defining events between discordant individuals compared to non-discordant,^1^ or reporting an association after combining non-AIDS with AIDS-defining events as the outcome.^25^

We conducted this study in a large, diverse cohort of adults receiving HIV care throughout several sub regions in Latin America. We used datasets that are frequently audited to ensure confidence in all clinical data. In addition, we analyzed data using Cox models for time-varying variables, using only the information of each specific period of time between consecutive CD4 counts measurements, without the need to make assumptions about future or interpolated values.^17^

We used the percentage time with CD4 <200, accounting for the total cumulative follow-up time with consistent viral suppression and focusing on the duration of time a patient spent in this condition. In this way, our measure does not make trapezoidal rule assumptions which could imply that individuals with very different CD4 counts may have the same cumulative immunologic responses so long as the total area under the curve of their CD4 trajectories is the same.^11^ Accounting for fluctuations in CD4 counts over time without asserting the equivalence of these cumulative measures, as our method does, is less likely to obscure the fact that lower individual CD4 counts may be associated with poorer outcomes. Moreover, it could be easier, in routine clinical practice to calculate a cumulative percentage over time compared to other measures requiring techniques such as trapezoid methods.

Our study had several limitations. We included all the events reported after ART initiation and viral suppression at the start of the follow-up period, but we were not able to identify which specific AIDS-defining or serious non-AIDS defining events might have been prevalent outcomes; though this may have caused some measurement error, it ensured that we included all incident outcomes, because comorbidity diagnosis is dependent on clinical judgment and local resources. As we have documented heterogeneous frequency of CD4 count measurements in the cohort,^26^ we have allowed CD4 counts to be carried forward up until 12 months; this represents the real practice in our region but could impact our results. Yet, the median days between consecutive CD4 counts was 156 days (IQR: 101-196) reducing the possibility of misclassification of periods of time with low CD4 counts. A recent study found a decrease in the frequency of pre-ART CD4 count measurements after Treat All policy adoption, especially in low/middle income countries, and suggested more research is needed to understand its effect on opportunistic infection prophylaxis management.^27^ In spite of current recommendations to perform less CD4 monitoring in virological suppressed patients,^28^ our study supports the importance of continued CD4 count monitoring to identify patients with a higher risk of developing AIDS-defining events or serious non-AIDS-defining events, in particular in those patients with CD4 counts lower than 200 cells/mm^3^. Finally, our data come from specific clinical cohorts in each site and could be non-representative of their respective countries.

## Conclusion

The association between higher cumulative percentage of time with low CD4 counts and poor outcomes of mortality and clinical events suggest that these patients require more screening for early diagnosis of comorbidities and perhaps different prophylactic practices, even if they remain virally suppressed.

## Contributors

YCV, BES, PR, CM, PB and JSM, contributed to study conception, data analysis and interpretation, and writing the first draft. YCV, BES and PR also contributed to statistical analysis and verified the underlying data. All authors contributed equally to critical review and approved the final manuscript.

## Funding

This work was supported by the NIH-funded Caribbean, Central and South America network for HIV epidemiology (CCASAnet, U01AI069923), a member cohort of the International Epidemiologic Databases to Evaluate AIDS (leDEA). This award is funded by the following institutes: Eunice Kennedy Shriver National Institute Of Child Health & Human Development (NICHD), National Cancer Institute (NCI), National Institute Of Allergy And Infectious Diseases (NIAID), National Institute Of Mental Health (NIMH), the National Heart, Lung, and Blood Institute (NHLBI), the National Institute on Alcohol Abuse and Alcoholism (NIAAA), the National Institute of Diabetes and Digestive and Kidney Diseases (NIDDK), and the Fogarty International Center (FIC). Specific funding was provided from the Fogarty International Center (FIC) for lead author, Yanink Caro-Vega, for the Fogarty-IeDEA Mentorship Program (FIMP).

## Data sharing statement

Data are made available to all authors and external collaborators upon reasonable request, in accordance with internal scientific review guidelines (https://www.ccasanet.org/collaborate/). External requests should be directed to CCASAnet Program Coordinator, Ms. Hilary Vansell (hilary.vansell@vumc.org).

## Declaration of interests

YCV, PR, BES, CC, CPC, CM and JSM declare payments made to their institutions from a NIH/NIDA Grant. PR and CPC declare receive consulting fees no related to this manuscript. PR has participated on a Data Safety Monitoring Board. PB, BCR, PM, DP, EG declare no have conflict of interest.

## Appendix Supplementary materials

Image, application 1

Image, application 2
