# Supplementary material for: Clinical effects of durability of immunosuppression in virologically suppressed ART-initiating persons with HIV in Latin America. A retrospective cohort study
Source: Lancet Reg Health Am. 2022 Jan 13;8:100175. doi: 10.1016/j.lana.2021.100175 (PMC9121860; doi:10.1016/j.lana.2021.100175)
Supplement: Supplementary file 1 [file mmc1.docx]

**Captions for Supplementary Material**

**Supplementary Figure 1.** Description of follow-up of the patients included in the study

**Note:** The follow-up of a patient ended:

1. At the time of the first viral load equals or higher than 200copies/mL.

2. If a patient had a last viral load lower than 200 and no other viral measurement in the next 365 days, there are two options:

2A. His/her last alive date occurs during less than 365 days, and then he/she finished his follow-up at last alive date.

2B. His/her last alive date occurs after 365 days, and then he/she finished his/her follow-up at last VL available.

**Supplementary Figure 2**. Number of patients per time since start of follow-up (that is, time in observation), under viral suppression (red) and, among these, those with CD4 count <200 (in blue). Panel A shows the distribution for patients initiating ART with CD4 cell count <200 and Panel B for those with CD4≥200.

**Note:** Panel A shows the group starting ART with CD4 less than 200. In red the number of patients under viral suppression and in blue patients with CD4 less than 200. Panel B shows the same areas for the group starting ART with CD4 higher than 200

**Supplementary Table 1. Distribution of ADE and SNADE.**

**Note:** Among 584 patients, we observed 618 ADE and SNADE, 409 ADE and 209 SNADE. A total of 375 patients had only ADE, 175 only SNADE and 34 had both of outcomes. Distribution of patients by type of ADE and SNADE is shown in the table below.

**Supplementary Table 2. Factors associated to higher percentage of time of CD4 count below 200.**

**Note:** To identify sociodemographic, clinical and laboratory factors at cART initiation associated to higher percentage time of CD4 count below 200 we used a logistic model including age, sex, route of transmission and clinical site.
