# Supplementary material for: Clinical effects of durability of immunosuppression in virologically suppressed ART-initiating persons with HIV in Latin America. A retrospective cohort study
Source: Lancet Reg Health Am. 2022 Jan 13;8:100175. doi: 10.1016/j.lana.2021.100175 (PMC9121860; doi:10.1016/j.lana.2021.100175)
Supplement: Supplementary file 2 [file mmc2.docx]

**Supplementary Tables**

**Supplementary Table 1. Distribution of ADE and SNADE.**

| **Type of event** | | **Number of patients (%)** |
| --- | --- | --- |
| ADE, n=409 |  |  |
|  | Cancer | 84 (20) |
|  | Candidiasis | 34 (8.3) |
|  | Cryptococcosis | 14 (3.4) |
|  | Cytomegalovirus | 13 (3.2) |
|  | Encephalopaty | 4 (<1) |
|  | Herpes | 17 (4.1) |
|  | Histoplasmosis | 4 (<1) |
|  | Leukoencephalopaty | 2 (<1) |
|  | Mycobacterium | 5 (1.2) |
|  | Pneumocystis | 27 (6.6) |
|  | Pneumonia | 19 (4.6) |
|  | Retinitis | 5 (1.2) |
|  | Salmonella | 2 (<1) |
|  | Toxoplasmosis | 19 (4.6) |
|  | Tuberculosis | 149 (36.4) |
|  | Wasting | 17 (4.1) |
| SNADE, n=209 |  |  |
|  | Cancer | 136 (65) |
|  | Cardiovascular | 31 (14.8) |
|  | Cerebrovascular | 12 (5.7) |
|  | Coronary | 3 (1.4) |
|  | Liver | 11(5.3) |
|  | Renal | 16 (7.6) |

**Note:** Among 584 patients, we observed 618 ADE and SNADE, 409 ADE and 209 SNADE. A total of 375 patients had only ADE, 175 only SNADE and 34 had both of outcomes. Distribution of patients by type of ADE and SNADE is shown in the table below.

**Supplementary Table 2. Factors associated to higher percentage of time of CD4 count below 200.**

|  | **OR** | **95%CI** | **p-value** |
| --- | --- | --- | --- |
| **Male** | 1.57 | 1.29 – 1.91 | <0.001 |
| **Age at Enrol (10 years)** | 1.18 | 1.10 – 1.26 | <0.001 |
| **Probable HIV acquisition risk** |  |  |  |
| Heterosexual | 1 |  |  |
| Men who have sex with Men | 0.79 | 0.66 – 0.94 | 0.007 |
| Other | 1.42 | 0.75 – 2.69 | 0.28 |
| Unknown | 1.11 | 0.82 – 1.51 | 0.49 |
| **Less than 200 at ART initiation** | 2.62 | 1.93 – 3.55 | <0.001 |
| **Site** |  |  |  |
| Argentina | 1 |  |  |
| Brazil | 0.85 | 0.57 – 1.29 | 0.46 |
| Chile | 1.39 | 0.94 – 2.07 | 0.09 |
| Honduras | 1.85 | 1.19 – 2.87 | 0.005 |
| Mexico | 1.20 | 0.79 – 1.82 | 0.39 |
| Peru | 1.03 | 0.68 – 1.55 | 0.87 |
| **Education level** |  |  |  |
| Primary | 1 |  |  |
| Secondary | 0.89 | 0.70 – 1.13 | 0.36 |
| University | 0.72 | 0.55 – 0.94 | 0.02 |
| Unknown | 1.05 | 0.74 – 1.49 | 0.78 |
| **Calendar year of follow-up start** | 1.02 | 1.00 – 1.04 | 0.004 |

**Note:** To identify sociodemographic, clinical and laboratory factors at cART initiation associated to higher percentage time of CD4 count below 200 we used a logistic model including age, sex, route of transmission and clinical site.
